# Should gestational weight gain charts exclude individuals with excess postpartum weight retention?

**DOI:** 10.1111/jhn.13310

**Published:** 2024-04-23

**Authors:** Peter M. Socha, Kari Johansson, Lisa M. Bodnar, Jennifer A. Hutcheon

**Affiliations:** 1 Department of Epidemiology, Biostatistics and Occupational Health, McGill University, Montreal, QC, Canada; 2 Clinical Epidemiology Division, Department of Medicine Solna, Karolinska Institutet, Solna, Sweden; 3 Department of Epidemiology, School of Public Health, University of Pittsburgh, Pittsburgh, PA, USA; 4 Department of Obstetrics and Gynaecology, University of British Columbia, Vancouver, BC, Canada

**Keywords:** Charts, gestational weight gain, interpregnancy weight, postpartum weight retention, recommendations, standards

## Abstract

**Background:**

High gestational weight gain is associated with excess postpartum weight retention, yet excess postpartum weight retention is not an exclusion criterion for current gestational weight gain charts. We aimed to assess the impact of excluding individuals with high interpregnancy weight change (a proxy for excess postpartum weight retention) on gestational weight gain distributions.

**Methods:**

We included individuals with an index birth from 2008 to 2014 and a subsequent birth before 2019, in the population-based Stockholm-Gotland Perinatal Cohort. We estimated gestational weight gain (kg) at 25 and 37 weeks, using weight at first prenatal visit (<14 weeks) as the reference. We calculated high interpregnancy weight change (≥10 kg and ≥5 kg) using the difference between weight at the start of an index and subsequent pregnancy. We compared gestational weight gain distributions and percentiles (stratified by early-pregnancy body mass index) before and after excluding participants with high interpregnancy weight change.

**Results:**

Among 55,723 participants, 17% had ≥10 kg and 34% had ≥5 kg interpregnancy weight change. The third, tenth, 50th, 90th and 97th percentiles of gestational weight gain were similar (largely within 1 kg) before versus after excluding participants with high interpregnancy weight change, at both 25 and 37 weeks. For example, among normal weight participants at 37 weeks, the 50th and 97th percentiles were 14 kg and 23 kg including versus 13 kg and 23 kg excluding participants with ≥5 kg interpregnancy weight change.

**Conclusions:**

Excluding individuals with excess postpartum weight retention from normative gestational weight gain charts may not meaningfully impact the charts’ percentiles.

## INTRODUCTION

High or low gestational weight gain is associated with adverse maternal and infant outcomes.^1–7^ The current World Health Organization (WHO) recommendations for optimal gestational weight gain cite the US National Academy of Medicine (NAM; formerly Institute of Medicine [IOM]) guidelines, but the NAM guidelines were developed for the USA and may not generalize to a global context, particularly to low- and middle-income countries.^8,9^ Therefore, the WHO is developing new global standards for gestational weight gain.^10^

Gestational weight gain standards are prescriptive charts that describe patterns of gestational weight gain under optimal circumstances. Gestational weight gain standards should therefore exclude individuals with factors that are causes, indicators or consequences of insufficient or excess gestational weight gain.^11–13^ For example, recent Swedish gestational weight gain charts excluded individuals with anomalous births, preterm births, pre-existing hypertension and pre-existing type I or II diabetes.^11^ Standards are distinct from reference charts, which simply describe the patterns of gestational weight gain in a population. Reference charts should not be used clinically to recommend how much weight an individual ought to gain during pregnancy because they include gestational weight gain values from individuals with diseases or other factor that caused them to gain too much (or too little) weight.

Excess postpartum weight retention is both strongly associated with high gestational weight gain and affects a large proportion of pregnant people.^4,5,7^ Including individuals with excess postpartum weight retention in gestational weight gain standards may therefore shift the charts’ percentiles to higher values that are not optimal for maternal health, but are instead associated with increased risks of excess postpartum weight retention. Nevertheless, existing charts have not excluded individuals with excess postpartum weight retention.^11–16^ This is likely because data on postpartum weight retention require regular postpartum follow-up data, which are expensive and time-consuming to collect. Therefore, a key question for new gestational weight gain standards is whether regular postpartum follow-up data should be collected to exclude individuals with excess postpartum weight retention.^17^

Our objective was to determine whether excluding individuals with excess postpartum weight retention impacts the percentile values of normative gestational weight gain charts, by comparing the distribution of gestational weight gain at specific weeks of gestation.

## METHODS

### Study population

We used population-based data from the Stockholm-Gotland perinatal cohort, which contains electronic medical records of all prenatal visits and delivery admissions for pregnant individuals in the Stockholm and Gotland counties of Sweden.^18^ We defined our study population using the same criteria used to derive previous Swedish gestational weight gain charts, restricting to births from January 2008 to October 2014, and excluding participants with multiple gestations, pre-existing hypertension, pre-existing type 1 or type 2 diabetes, preterm or postterm birth (<37 weeks or >41 weeks; as determined by date of embryo transfer or ultrasound-confirmed estimate), congenital anomalies and stillbirth.^11^ We used the unique personal identification number assigned to each Swedish resident to link participants to all subsequent births (≥22 weeks gestational age) through October 2019, and restricted our study population to participants with at least one subsequent birth.

### Body mass index

We estimated early-pregnancy body mass index (BMI; kg/m^2^) using the first measured weight in early-pregnancy (<14 weeks) and self-reported height. Participants were classified into underweight (<18.5 kg/m^2^), normal weight (18.5–24.9 kg/m^2^), overweight (25–29.9 kg/m^2^) and obese (≥30 kg/m^2^).

### Gestational weight gain

Weight measurements (rounded to the nearest kg) are routinely measured and recorded by health professionals during prenatal visits in Sweden. We excluded implausible early-pregnancy weights, defined as >350 kg or <30 kg, or gestational weight gain >6 SDs from the mean at that week of gestation or (among individuals with a minimum of five weight observations) >6 SDs from the weight expected given the individual’s past weight.^19^ Data on pre-pregnancy weight were unavailable in our cohort, and so we defined gestational weight gain as the difference between a participants’ weight at their first prenatal visit (<14 weeks) and weight at each subsequent prenatal visit.

### Interpregnancy weight change

We used interpregnancy weight change (the difference between weight at the start of the index and subsequent pregnancies) as a proxy for post-partum weight retention because data on postpartum weight retention are unavailable in the Stockholm-Gotland Perinatal Cohort. We categorised interpregnancy weight change in two ways: ≥10 kg and ≥5 kg. These categories have been used in a previous study on the association between gestational weight gain and interpregnancy weight change.^3^

### Analysis

We sought to examine the impact of excluding individuals with high interpregnancy weight change on weight gain percentiles at different time points during pregnancy. We therefore selected a week of gestation from each of the second and third trimesters for analysis, using the week that had the greatest number of gestational weight gain measurements available in each trimester. This approach was chosen to maximise our sample size and ability to detect differences in distributions.

At each of these weeks, we compared the mean gestational weight gain in participants with high interpregnancy weight change to the mean gestational weight gain in those without. We then plotted the distribution of gestational weight gain at each of these weeks, and calculated gestational weight gain percentiles (third, tenth, 50th, 90th and 97th) with 95% confidence intervals (CIs), before and after excluding participants with high interpregnancy weight change. We stratified results by early-pregnancy BMI, as is commonly performed for gestational weight gain charts.^13^

### Sensitivity analysis

By using interpregnancy weight change as a proxy for excess post-partum weight retention, we necessarily limited our analyses to individuals who went on to have a subsequent pregnancy. If weight gain patterns among individuals with a subsequent pregnancy differ from those in individuals without a subsequent pregnancy, this could limit the generalisability of our findings to standards intended for use among all pregnancies. To examine whether restricting to participants with a subsequent pregnancy during our follow-up period limited the generalisability of our results, we compared the distributions of gestational weight gain before and after restricting to participants with at least one subsequent pregnancy (among participants who met all other inclusion criteria).

## RESULTS

Forty-three percent (65,484/151,796) of individuals in the Stockholm-Gotland perinatal cohort had at least one subsequent pregnancy before the end of follow-up (October 2019), 85% percent (55,723/65,484) of whom met the additional inclusion criteria for our study population. The frequency of weight gain measurements across gestational age (in weeks) are presented in the Supporting information (Figure S1). The later-pregnancy week with the most gestational weight gain measurements was 37 weeks (*N* = 20,548; 37% of study population) and the earlier-pregnancy period with the most gestational weight gain measurements was 25 weeks (*N* = 16,660; 30% of study population). Study population characteristics stratified by BMI are presented for age, parity at index birth, gestational weight gain, interpregancy interval, and interpregnancy weight change in the Supporting information (Table S1).

Mean gestational weight gain was 14 kg at 37 weeks and 7.0 kg at 25 weeks (see Supporting information, Table S1). High interpregnancy weight change was common and was relatively consistent across BMI categories, with 17% of the participants’ weight increasing by ≥10 kg and 34% increasing by ≥5 kg (Table 1). At 37 weeks, gestational weight gain was 1–3 kg higher (across BMI groups) among individuals with high interpregnancy weight change compared to their counterparts with lower interpregnancy weight change. Differences in gestational weight gain at 25 weeks between groups were <1 kg higher (across BMI groups), although the absolute amount of weight gain at this period of gestation was also lower.

**FIGURE 1:**
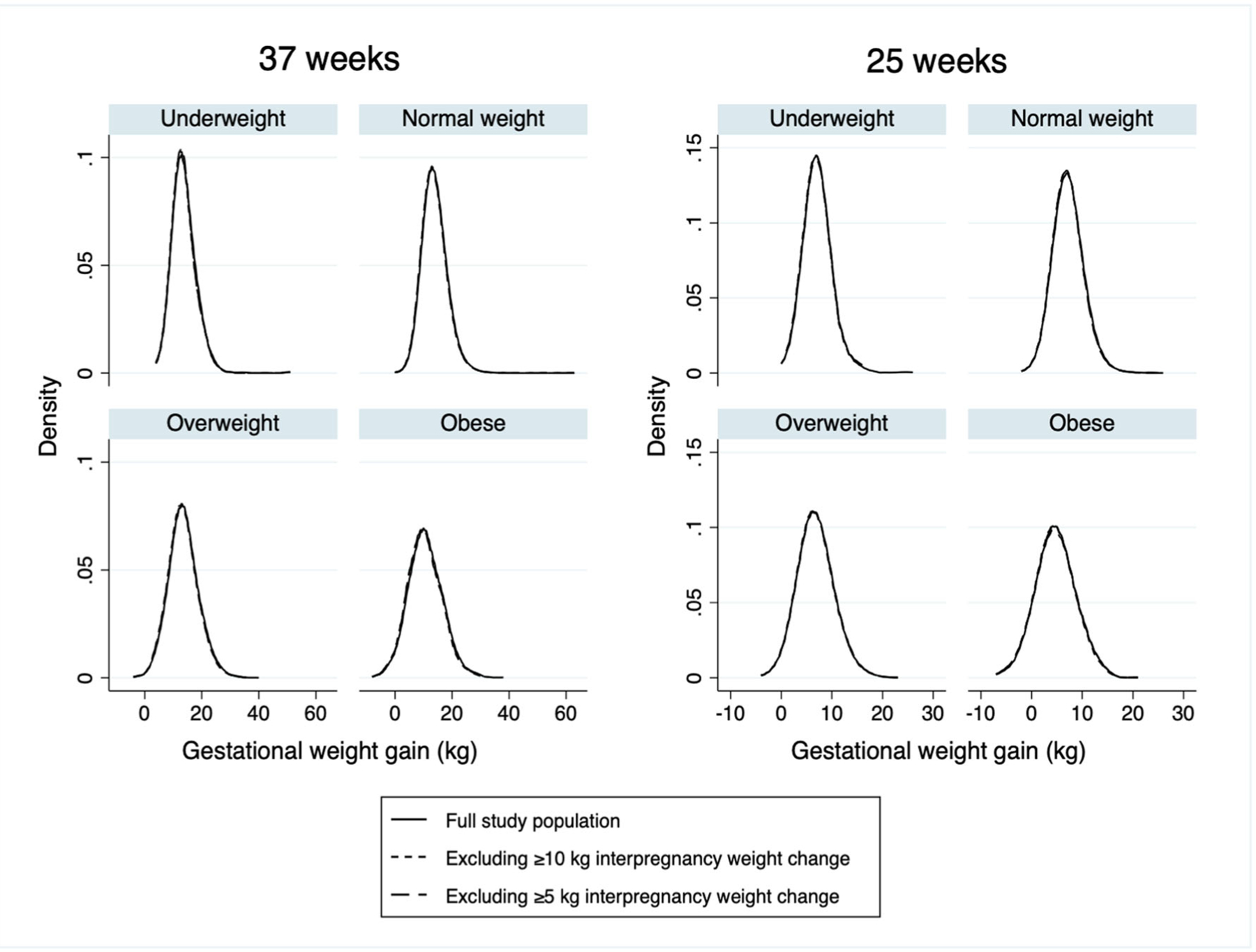
Distribution of gestational weight gain at 37 (*N* = 20,548) and 25 (*N* = 16,660) weeks gestational age among participants in the Stockholm-Gotland perinatal cohort with an index pregnancy between 2008 and 2014 and a subsequent pregnancy through 2019, before and after excluding participants with high interpregnancy weight change.

Despite the differences in mean gestational weight gain between participants with versus without high interpregnancy weight change, the distributions of gestational weight gain at 37 weeks and 25 weeks were very similar before and after excluding participants with high interpregnancy weight change (Figure 1). Of the 40 calculated percentiles (5 percentiles × 4 BMI categories × 2 visits), 31 (78%) had the same point estimate when excluding participants with either ≥10 kg or ≥5 kg interpregnancy weigh change (see Supporting information, Table S2). Of the nine percentiles (23%) that had a different point estimate, all were within 1 kg and only three (7.5%) had confidence intervals that did not overlap.

Sensitivity analyses found that gestational weight gain distributions at 37 and 25 weeks were very similar before and after excluding individuals who would have met inclusion criteria but did not have a subsequent pregnancy through October 2019 (see Supporting information, Figures S2 and S3).

## DISCUSSION

We found that excluding participants with high interpregnancy weight change did not meaningfully impact gestational weight gain distributions or percentiles at 37 and 25 weeks, in a population-based cohort of pregnant individuals in two Swedish counties.

Our results support the use of the current Swedish gestational weight gain charts, which do not exclude individuals with excess postpartum weight retention.^11^ Whether our results generalise to support the use of charts in other regions depends largely on whether the patterns of gestational weight gain, interpregnancy weight change and postpartum weight retention are similar to Sweden.^12–16^ The amount of postpartum weight retention and the association with gestational weight gain can differ between regions, and it is possible that differences in gestational weight gain percentiles before and after excluding participants with high interpregnancy weight change would be more pronounced in populations where (1) the mean interpregnancy weight change is higher or (2) the difference in gestational weight gain between participants with versus without interpregnancy weight change is larger.^20,21^ These same considerations apply to the development of future charts, where the collection of information on postpartum weight retention also has pragmatic implications as routine postpartum follow-up data are rare and can be expensive and time-consuming to collect. Our results do not have direct implications for gestational weight gain recommendations that are based on identifying the range of gestational weight gain values that minimise adverse health outcomes (rather than standard charts), such as the NAM guidelines.

Given the positive association between gestational weight gain and postpartum weight retention, we had expected to see gestational weight gain distributions shifted lower (and reference values to be correspondingly smaller) when excluding participants with high interpregnancy weight change.^4,5,7^ To change the distribution or percentiles of gestational weight gain, exclusion criteria need to be both strongly associated with gestational weight gain and common. Because high interpregnancy weight change was relatively common in our cohort, our results likely reflect that the association between gestational weight gain and interpregnancy weight change not being strong enough. Simulation studies would be particularly useful for investigating how gestational weight gain distributions may, or may not, change based on the strength of the associations and/or the distributions of gestational weight gain and postpartum weight retention.

The strengths of our study are that the cohort is population-based and that weight measurements were routinely collected during prenatal visits, reducing the likelihood of selection bias. Potential limitations of our study include missing data, as not all participants had a prenatal visit at exactly 25 or 37 weeks. However, because the exact week of prenatal visits is somewhat arbitrary, we have no reason to assume that participants who had weight measurements at exactly these weeks were meaningfully different than participants that who had weight measurements shortly before or after 25 and 37 weeks. Second, we used interpregnancy weight change as a proxy for postpartum weight retention because we did not have routinely collected postpartum weight measurements. Interpregnancy weight change is an imperfect proxy for postpartum weight retention because the change in weight could be the result of weight gained after the delivery of the index birth rather than weight retained from the index pregnancy. However, high interpregnancy weight change would still capture the participants with the most consistent postpartum weight retention (i.e., participants who retained their weight postpartum and did not lose weight before their next pregnancy). At the same time, interpregnancy weight change may be an important potential exclusion criteria for gestational weight gain charts in itself, as it has been previously been linked to high gestational weight gain and adverse perinatal outcomes in subsequent pregnancies.^3,6,22^ Finally, excluding participants without a subsequent pregnancy could limit the generalisability of our results, although it was reassuring that gestational weight gain distributions were similar before and after excluding pregnant individuals who did not have a subsequent pregnancy.

## CONCLUSIONS

Despite the positive association between gestational weight gain and postpartum weight retention, excluding individuals with excess postpartum weight retention did not meaningfully impact normative values of gestational weight gain charts in our cohort. However, the generalisability of our results should be considered, particularly for chart development in populations with higher postpartum weight retention or stronger associations between gestational weight gain and postpartum weigh retention.

## Supplementary Material

supplement

## Figures and Tables

**TABLE 1:** Mean gestational weight gain (kg) among *N* = 55,723 participants in the Stockholm-Gotland perinatal cohort with an index pregnancy between 2008 and 2014 and a subsequent pregnancy through 2019, who did versus did not have high interpregnancy weight change.

	37 weeks		25 weeks	
		
Interpregnancy weight gain	Gestational weight gain	N (%)	Gestational weight gain	N (%)
Overall				
<10 kg	13 ± 4.6	17,266 (84)	6.9 ± 3.1	13,880 (83)
≥10 kg	15 ± 4.7	3282 (16)	7.6 ± 3.2	2780 (17)
<5 kg	13 ± 4.6	13,927 (68)	6.9 ± 3.1	10,958 (66)
≥5 kg	14 ± 4.7	6621 (32)	7.2 ± 3.1	5693 (34)
Underweight				
<10 kg	13 ± 3.9	608 (86)	7.0 ± 2.5	499 (85)
≥10 kg	16 ± 5.5	103 (14)	8.0 ± 3.8	85 (15)
<5 kg	13 ± 3.9	481 (68)	7.0 ± 2.4	395 (68)
≥5 kg	15 ± 4.7	230 (32)	7.6 ± 3.3	189 (32)
Normal weight				
<10 kg	14 ± 4.2	12,173 (85)	7.1 ± 2.8	9648 (84)
≥10 kg	15 ± 4.2	2217 (15)	7.8 ± 2.9	1863 (16)
<5 kg	14 ± 4.3	9816 (68)	7.2 ± 2.8	7662 (67)
≥5 kg	14 ± 4.2	4574 (32)	7.4 ± 2.8	3849 (33)
Overweight				
<10 kg	13 ± 5.1	3358 (84)	6.7 ± 3.5	2756 (82)
≥10 kg	15 ± 5.4	660 (16)	7.5 ± 3.4	617 (18)
<5 kg	13 ± 5.2	2674 (67)	6.7 ± 3.5	2174 (64)
≥5 kg	14 ± 5.2	1344 (33)	7.1 ± 3.5	1199 (36)
Obese				
<10 kg	10 ± 6.0	1199 (84)	4.7 ± 3.8	1002 (84)
≥10 kg	12 ± 5.9	229 (16)	5.3 ± 3.7	190 (16)
<5 kg	11 ± 6.1	956 (67)	4.7 ± 3.9	792 (66)
≥5 kg	11 ± 5.8	472 (33)	4.9 ± 3.7	400 (34)

*Note*: Data are reported as the mean ± SD.

## Data Availability

The data that support the findings of this study are from the Stockholm–Gotland Perinatal Cohort. Restrictions apply to the availability of these data, which were used under license for the present study.

## Key points

- Excess postpartum weight retention is strongly associated with high gestational weight gain, yet it is not an exclusion criterion for current gestational weight gain charts.
- We found that excluding individuals with high interpregnancy weight change did not impact the distribution or percentiles of gestational weight gain at 25 or 37 weeks, in a Swedish population-based birth cohort.

## References

[1] Voerman E, Santos S, Inskip H, Amiano P, Barros H, Charles MA, Association of gestational weight gain with adverse maternal and infant outcomes. JAMA. 2019;321(17): 1702–15. 10.1001/jama.2019.3820 31063572 PMC6506886

[2] Goldstein RF, Abell SK, Ranasinha S, Misso M, Boyle JA, Black MH, Association of gestational weight gain with maternal and infant outcomes: a systematic review and meta-analysis. JAMA. 2017;317(21):2207–25. 10.1001/jama.2017.3635 28586887 PMC5815056

[3] Hutcheon JA, Chapinal N, Bodnar LM, Lee L. The INTERGROWTH-21st gestational weight gain standard and interpregnancy weight increase: a population-based study of successive pregnancies. Obesity. 2017;25(6):1122–7. 10.1002/oby.21858 28474509 PMC5488248

[4] Mannan M, Doi SA, Mamun AA. Association between weight gain during pregnancy and postpartum weight retention and obesity: a bias-adjusted meta-analysis. Nutr Res 2013;71(6):343–52. 10.1111/nure.12034 23731445

[5] Rong K, Yu K, Han X, Szeto IM, Qin X, Wang J, Pre-pregnancy BMI, gestational weight gain and postpartum weight retention: a meta-analysis of observational studies. Public Health Nutr 2015;18(12):2172–82. 10.1017/S1368980014002523 25411780 PMC10271485

[6] Whelan E, Armson BA, Ashley-Martin J, MacSween K, Woolcott C. Gestational weight gain and interpregnancy weight change in adolescent mothers. J Pediatr Adolesc Gynecol 2017;30(3):356–61. 10.1016/j.jpag.2017.02.006 28274683

[7] Carrilho TRB, Hutcheon JA, Rasmussen KM, Reichenheim ME, Farias DR, Freitas-Costa NC, Gestational weight gain according to the Brazilian charts and its association with maternal and infant adverse outcomes. Am J Clin Nutr 2023;117(2):414–25. 10.1016/j.ajcnut.2022.11.021 36811564

[8] Committee to reexamine IOM pregnancy weight guidelines food and nutrition board, board on children, youth, and families. In: Weight Gain During Pregnancy: Reexamining the Guidelines. Institute of Medicine; 2009.

[9] World Health Organization. WHO Recommendations on Antenatal Care for a Positive Pregnancy Experience. World Health Organization; 2016. Available from: https://www.who.int/publications/i/item/9789241549912 28079998

[10] The First Meeting of the WHO Technical Advisory Group on Gestational Weight Gain (TAG-GWG). World Health Organization; 2023. Available from: https://www.who.int/publications/m/item/report-on-the-first-meeting-of-the-who-technical-advisory-group-on-gestational-weight-gain-(tag-gwg)

[11] Johansson K, Hutcheon JA, Stephansson O, Cnattingius S. Pregnancy weight gain by gestational age and BMI in Sweden: a population-based cohort study. Am J Clin Nutr 2016;103(5): 1278–84. 10.3945/ajcn.115.110197 27009753

[12] Santos S, Eekhout I, Voerman E, Gaillard R, Barros H, Charles MA, Gestational weight gain charts for different body mass index groups for women in Europe, North America, and Oceania. BMC Med 2018;16(1):201. 10.1186/s12916-018-1189-1 30396358 PMC6217770

[13] Ohadike CO, Cheikh-Ismail L, Ohuma EO, Giuliani F, Bishop D, Kac G, Systematic review of the methodological quality of studies aimed at creating gestational weight gain charts. Adv Nutr 2016;7(2):313–22. 10.3945/an.115.010413 26980814 PMC4785472

[14] Cheikh Ismail L, Bishop DC, Pang R, Ohuma EO, Kac G, Abrams B, Gestational weight gain standards based on women enrolled in the Fetal Growth Longitudinal Study of the INTERGROWTH-21st Project: a prospective longitudinal cohort study. BMJ 2016;352:i555. 10.1136/bmj.i555 26926301 PMC4770850

[15] Kac G, Carilho TR, Rasmussen KM, Reichenheim ME, Farias DR, Hutcheon JA. Gestational weight gain charts: results from the Brazilian Maternal and Child Nutrition Consortium. Am J Clin Nutr 2021;113(5):1351–60. 10.1093/ajcn/nqaa402 33740055 PMC8106749

[16] Huang A, Xiao Y, Hu H, Zhao W, Yang Q, Ma W, Gestational weight gain charts by gestational age and body mass index for chinese women: a population-based follow-up study. J Epidemiol 2020;30(8):345–53. 10.2188/jea.JE20180238 31474675 PMC7348073

[17] GWG Steering Committee. Global gestational weight gain standards project: eligibility criteria for the inclusion of study datasets into the pooled underlying database. World Health Organization; 2023. Available from: https://cdn.who.int/media/docs/default-source/nutrition-and-food-safety/technical-advisory-group-on-gestational-weight-gain/tag-gwg-annex3-study-level-eligibilitycriteria.pdf

[18] Johansson K, Granfors M, Petersson G, Bolk J, Altman M, Cnattingius S, The Stockholm–Gotland perinatal cohort—a population-based cohort including longitudinal data throughout pregnancy and the postpartum period. Paediatr Perinat Epidemiol 2023;37(4):276–86. 10.1111/ppe.12945 36560891

[19] Boone-Heinonen J, Tillotson CJ, O’Malley JP, Marino M, Andrea SB, Brickman A, Not so implausible: impact of longitudinal assessment of implausible anthropometric measures on obesity prevalence and weight change in children and adolescents. Ann Epidemiol 2019;31:69–74.e5. 10.1016/j.annepidem.2019.01.006 30799202 PMC6450088

[20] Nehring I, Schmoll S, Beyerlein A, Hauner H, von Kries R. Gestational weight gain and long-term postpartum weight retention: a meta-analysis. Am J Clin Nutr 2011;94(5):1225–31. 10.3945/ajcn.111.015289 21918221

[21] Onyango AW, Nommsen-Rivers L, Siyam A, Borghi E, de Onis M, Garza C, Post-partum weight change patterns in the WHO Multicentre Growth Reference Study. Matern Child Nutr 2011;7(3):228–40. 10.1111/j.1740-8709.2010.00295.x 21338469 PMC6860679

[22] Timmermans YEG, van de Kant KDG, Oosterman EO, Spaanderman MEA, Villamor-Martinez E, Kleijnen J, The impact of interpregnancy weight change on perinatal outcomes in women and their children: a systematic review and meta-analysis. Obesity Rev 2019;21:e12974. 10.1111/obr.12974 PMC7050512 31751496

