# Supplementary material for: Should gestational weight gain charts exclude individuals with excess postpartum weight retention?
Source: J Hum Nutr Diet. Author manuscript; Available in PMC 2025 Aug 1. (PMC11771746; doi:10.1111/jhn.13310)
Supplement: supplement [file NIHMS2043002-supplement-supplement.docx]

**Supplement**

Socha PM, Johansson K, Bodnar LM, Hutcheon J. Should gestational weight gain charts exclude individuals with excess postpartum weight retention? 2024.

**Contents**

[Figure S1 2](#_Toc146361704)

[Figure S2 3](#_Toc146361705)

[Table S1 5](#_Toc146361706)

[Table S2 6](#_Toc146361707)


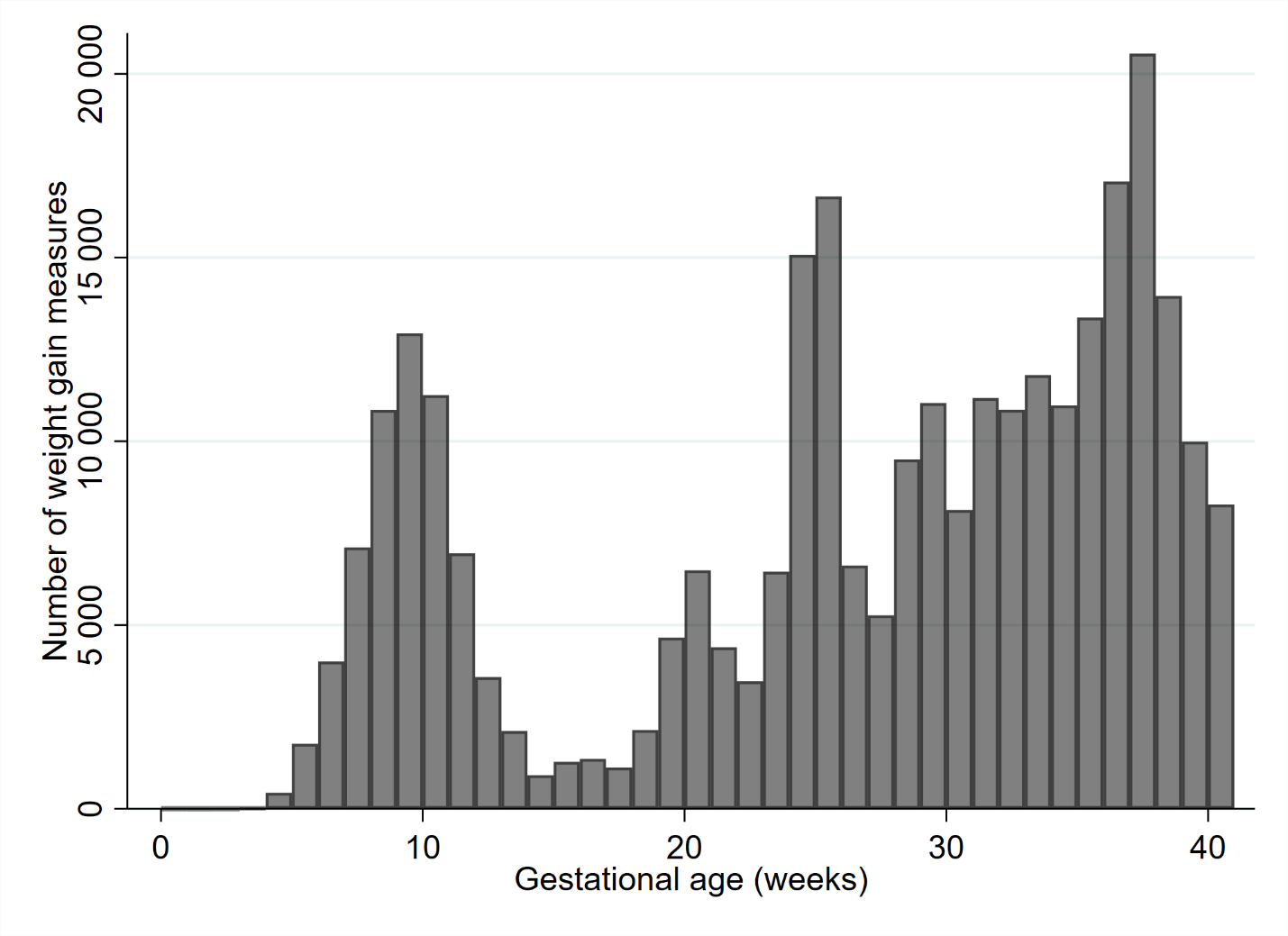


Figure S1. Frequency of gestational weight gain measurements by gestational age at visit among N=55,723 individuals in the Stockholm-Gotland perinatal cohort with an index pregnancy between 2008-2014 and a subsequent pregnancy through 2019.


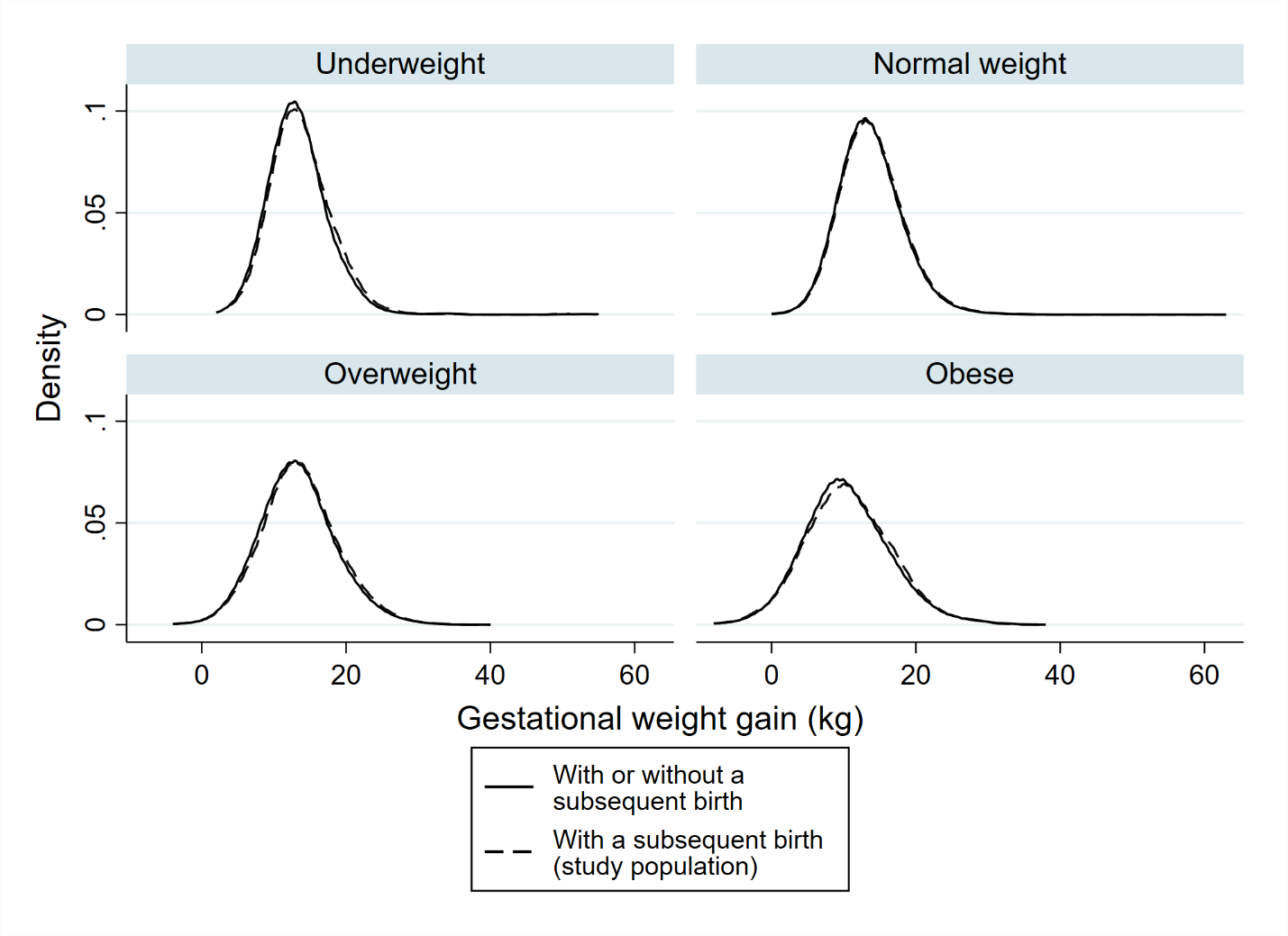


Figure S2. Distribution of gestational weight gain at 37 weeks among participants in the Stockholm-Gotland perinatal cohort with an index pregnancy from 2008-2014 before (N=45,408) and after (N=20,547) excluding participants who did not have a subsequent pregnancy through October 2019.


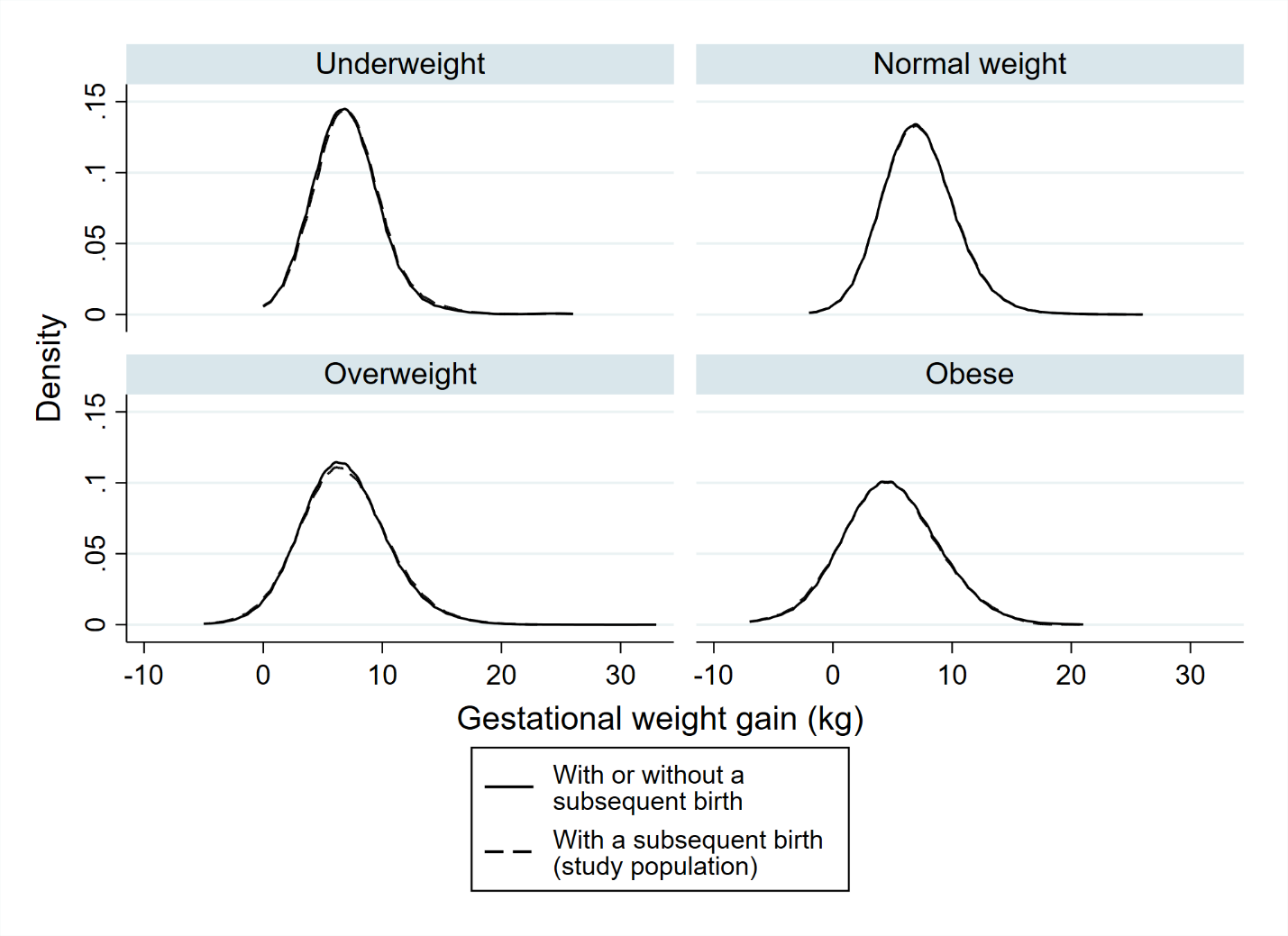
Figure S3. Distribution of gestational weight gain at 25 weeks among participants in the Stockholm-Gotland perinatal cohort with an index pregnancy from 2008-2014 before (N=37,740) and after (N=16,660) excluding participants who did not have a subsequent pregnancy through October 2019.

Table S1. Study population characteristics, stratified by early-pregnancy BMI. Data are from N=55,723 participants in the Stockholm-Gotland perinatal cohort with an index pregnancy from 2008-2014 and a subsequent pregnancy through 2019.

|  | | Underweight | Normal weight | Overweight | Obese | Overall |
| --- | --- | --- | --- | --- | --- | --- |
| N | | 1,949 (3.5) | 39,170 (70) | 10,713 (19) | 3,891 (7.0) | 55,723 (100) |
| Age at index birth (years) | | 28 ± 4.5 | 30 ± 4.3 | 29 ± 4.6 | 29 ± 4.7 | 30 ± 4.4 |
| Parity at index birth | |  |  |  |  |  |
|  | 1 | 1,434 (76) | 28,786 (73) | 6,843 (64) | 2,229 (57) | 39,292 (71) |
|  | 2 | 432 (22) | 8,416 (21) | 2,775 (26) | 1,053 (27) | 12,676 (23) |
|  | ≥3 | 83 (4.3) | 1,968 (5.0) | 1,095 (2.8) | 609 (16) | 3,755 (6.7) |
| Gestational weight gain (kg) | |  |  |  |  |  |
|  | 37 weeks^a^ | 14 ± 4.2 | 14 ± 4.2 | 14 ± 5.2 | 11 ± 6.0 | 14 ± 4.7 |
|  | 25 weeks^b^ | 7.2 ± 2.7 | 7.3 ± 2.8 | 6.8 ± 3.5 | 4.8 ± 3.8 | 7.0 ± 3.1 |
| Interpregnancy interval (years) | | 2.6 ± 1.7 | 2.4 ± 1.6 | 2.5 ± 1.7 | 2.6 ± 1.9 | 2.5 ± 1.6 |
| Interpregnancy weight change (kg) | | 1.7 ± 7.9 | 1.3 ± 8.6 | 1.7 ± 9.4 | 0.25 ± 11 | 1.3 ± 8.9 |
| ≥10 kg interpregnancy weight change | | 305 (16) | 6,525 (17) | 2,031 (19) | 664 (17) | 9,542 (17) |
| ≥5 kg interpregnancy weight change | | 640 (33) | 13,182 (34) | 3,923 (37) | 1,340 (34) | 19,058 (34) |

Note: Data are reported as mean ± sd or N (%).

^a^N=711 underweight, 14,391 normal weight, 4,018 overweight, 1,428 obese, 20,548 overall

^b^N=584 underweight, 11,511 normal weight, 3,373 overweight, 1,192 obese, 16,660 overall

Table S2. Gestational weight gain percentiles in kg (95% CI) among participants in the Stockholm-Gotland perinatal cohort with an index pregnancy between 2008-2014 and a subsequent pregnancy through 2019, before and after excluding participants with high interpregnancy weight change.

|  |  | 37 weeks gestation | | | | | | 25 weeks gestation | | | | | |
| --- | --- | --- | --- | --- | --- | --- | --- | --- | --- | --- | --- | --- | --- |
| Percentile | | 3rd | 10th | 50th | 90th | 97th | N | 3rd | 10th | 50th | 90th | 97th | N |
| Underweight | |  |  |  |  |  |  |  |  |  |  |  |  |
|  | Full study cohort | 7  (6, 8) | 9  (9, 9) | 13  (13, 14) | 19  (19, 20) | 22  (21, 24) | 711 | 3  (2, 3) | 4  (4, 5) | 7  (7, 7) | 10  (10, 11) | 13  (12, 14) | 584 |
|  | Excluding ≥10 kg | 7  (6, 8) | 9  (9, 9) | 13  (13, 14) | 19  (18, 20) | 22  (21, 23) | 616 | 3  (2, 3) | 4  (4, 4) | 7  (7, 7) | 10  (10, 11) | 13  (11, 14) | 492 |
|  | Excluding ≥5 kg | 7  (6, 8) | 9  (9, 10) | 13  (13, 14) | 19  (18, 20) | 22  (21, 24) | 487 | 2  (2, 3) | 4  (4, 4) | 7  (7, 7) | 10  (10, 11) | 13  (12, 14) | 389 |
| Normal weight | |  |  |  |  |  |  |  |  |  |  |  |  |
|  | Full study cohort | 7  (7, 7) | 9  (9, 9) | 14  (14, 14) | 19  (19, 19) | 23  (22, 23) | 14,391 | 3  (2, 3) | 4  (4, 4) | 7  (7, 7) | 11  (11, 11) | 13  (13, 13) | 11,511 |
|  | Excluding ≥10 kg | 7  (7, 7) | 9  (9, 9) | 13  (13, 13) | 19  (19, 19) | 22  (22, 23) | 12,141 | 2  (2, 3) | 4  (4, 4) | 7  (7, 7) | 11  (10, 11) | 13  (13, 13) | 9,634 |
|  | Excluding ≥5 kg | 7  (7, 7) | 9  (9, 9) | 13  (13, 13) | 19  (19, 19) | 23  (22, 23) | 9,849 | 2  (2, 3) | 4  (4, 4) | 7  (7, 7) | 11  (10, 11) | 13  (13, 13) | 7,633 |
| Overweight | |  |  |  |  |  |  |  |  |  |  |  |  |
|  | Full study cohort | 4  (4, 5) | 7  (7, 8) | 13  (13, 14) | 20  (20, 21) | 24  (24, 25) | 4,018 | 1  (0, 1) | 3  (2, 3) | 7  (7, 7) | 11  (11, 11) | 14  (13, 14) | 3,373 |
|  | Excluding ≥10 kg | 4  (4, 5) | 7  (7, 7) | 13  (13, 13) | 20  (20, 20) | 24  (23, 24) | 3310 | 1  (0, 1) | 3  (2, 3) | 7  (6, 7) | 11  (11, 11) | 14  (13, 14) | 2,764 |
|  | Excluding ≥5 kg | 4  (4, 5) | 7  (7, 7) | 13  (13, 13) | 20  (20, 21) | 24  (23, 25) | 2,654 | 0  (0, 1) | 3  (2, 3) | 7  (7, 7) | 11  (11, 12) | 14  (14, 14) | 2,161 |
| Obese | |  |  |  |  |  |  |  |  |  |  |  |  |
|  | Full study cohort | 0  (-1, 0) | 3  (3, 4) | 10  (10, 11) | 18  (18, 19) | 23  (21, 24) | 1,428 | -2  (-3, -2) | 0  (0, 0) | 5  (4, 5) | 10  (9, 10) | 12  (12, 13) | 1,192 |
|  | Excluding ≥10 kg | -1  (-1, 0) | 3  (3, 4) | 10  (10, 10) | 18  (17, 18) | 22  (21, 23) | 1,199 | -3  (-4, -2) | 0  (0, 0) | 5  (4, 5) | 9  (9, 10) | 12  (11, 13) | 990 |
|  | Excluding ≥5 kg | -1  (-2, 0) | 3  (3, 4) | 10  (10, 10) | 18  (17, 19) | 22  (21, 23) | 937 | -3  (-4, -2) | 0  (0, 0) | 5  (4, 5) | 10  (9, 10) | 12  (12, 13) | 775 |
